# A competitive photoelectrochemical immunosensor based on a CdS-induced signal amplification strategy for the ultrasensitive detection of dexamethasone

**DOI:** 10.1038/srep17945

**Published:** 2015-12-09

**Authors:** Xueping Wang, Tao Yan, Yan Li, Yixin Liu, Bin Du, Hongmin Ma, Qin Wei

**Affiliations:** 1 Key Laboratory of Chemical Sensing & Analysis in Universities of Shandong, School of Chemistry and Chemical Engineering, University of Jinan, Jinan 250022, PR China; 2 School of Resources and Environment, University of Jinan, Jinan 250022, PR China

## Abstract

A novel photoelectrochemical immunosensor based on the competitive strategy is proposed for the specific detection of dexamethasone (DXM). Graphitic carbon nitride coupled with bismuth sulfide are used as the sensing matrix for the immobilization of BSA-DXM on the electrode surface, while cadmium sulfide functionalized titanium dioxide (TiO_2_@CdS) is used as the photoelectric active labels of anti-DXM. Due to the perfect matching of energy levels between TiO_2_ and CdS, the *in situ* prepared composite labels show excellent photocurrent response under visible lights. The competitive binding of DXM in sample solutions and BSA-DXM on the electrode surface reduces the specific attachment of labels to the electrode, resulting in a decrease of the photocurrent intensity. Greatly enhanced sensitivity is achieved after the optimization of the detection conditions. Under the optimal detection condition, the well-designed immunosensor for DXM exhibits a low detection limit of 2 pg∙mL^−1^. Additionally, the proposed immunoassay system shows high specificity, good reproducibility and acceptable stability, which is also expected to become a promising platform for the detection of other small molecules.

In recent years, the veterinary drugs are widely used in livestocks and animal products for growth promotion, which is extremely harmful to human health. Dexamethasone (DXM) is a kind of synthetic adrenal cortical hormone drugs which has the effect of anti-inflammatory, anti-allergic and shock resistance^1^,^2^. However, it has been illegally used as growth promoting agents to obtain an economical benefit from increased muscle development^3^,^4^,^5^, and their residues in meat and other animal products have toxic consequences for human health^6^,^7^. Besides, it also has been added into certain banned drugs because of misusing as a doping agent in sports to enhance the performance^8^. Thus, developing a sensitive and rapid detection method for the determination of DXM is of great importance in food and pharmaceutical drugs research.

Photoelectrochemical (PEC) sensing is a newly developed assay, which has drawn ever growing interest due to its desirable advantages and attractive potential in biological analysis^9^,^10^,^11^,^12^. Coupling the light irradiation and electrochemical detection, the PEC immunosensor is very sensitive because of the low background signals^13^,^14^,^15^. The property of photoactive materials, which can transform the light input into current output, is a key factor to influence the analytical performances of the PEC immunosensors^16^,^17^. Graphite-like carbon nitride (g-C_3_N_4_), emerges as a new photoactive material and has attracted much attention because of its good stability and moderate band gap^18^,^19^. However, the photoelectric conversion ability of pure g-C_3_N_4_ is limited due to the low separation efficiency of photoinduced electron-hole pairs and narrow visible light response range^20^.

To overcome these drawbacks, it was proposed that g-C_3_N_4_ could be coupled with narrow bandgap semiconductor for enhancement of photoelectric conversion efficiency. Bismuth sulfide (Bi_2_S_3_) is a semiconductor with a band gap between 1.3 and 1.7 eV and exhibits a broad absorption in a large range^21^. The high absorption coefficient and the reasonable conversion efficiency of incident photon to electron make it an excellent candidate as a light-harvesting substrate for PEC applications^22^. Moreover, the synthesized Bi_2_S_3_ nanorods (NRs) owned the large specific surface and excellent fast and long distance electron transport capability. Based on this thought, g-C_3_N_4_ coupled with Bi_2_S_3_ as photoelectric active materials was proposed. According to the relative position of energy bands, the coupled system could reduce the recombination of photogenerated electron-hole pairs effectively^23^, resulting in enhanced photocurrent response of g-C_3_N_4_.

Titanium oxide (TiO_2_) is one of most promising semiconducting material for PEC sensing due to its attractive advantages of high abundance, low cost and nontoxicity^24^,^25^. In this work, nanoporous TiO_2_ was used as labels for loading *in situ* generated CdS, showing excellent photocurrent response under visible lights since the perfect matching of energy levels between them.

Herein, an innovative PEC immunosensor was fabricated for the sensitive and specific detection of DXM based on the competitive strategy showed in Fig. 1. The carboxylated g-C_3_N_4_ and Bi_2_S_3_ were assembled on the indium tin oxide (ITO) electrode in turns, followed by calcining in muffle to ensure materials adhesion to the electrode and obtain amount of active sites. Compared to pristine g-C_3_N_4_, carboxylated g-C_3_N_4_ exhibits good dispersibility which benefits to the stability of biosensor. Then BSA-DXM was immobilized on the transducer surface using chitosan (CS) as a linker. For the competitive binding assay, the mixture of different concentrations of free DXM and Cd^2+^@TiO_2_ loaded anti-DXM (Cd^2+^@TiO_2_-anti-DXM) was anchored onto the electrode by specific immunoreactions of antigen-antibody, a higher concentration of DXM led to less Cd^2+^@TiO_2_-anti-DXM bound to the sensor surface and therefore reduced the signal. Finally, for the *in situ* generation of CdS which could generate a photocurrent as a readout signal, Na_2_S was dropped on the modified electrode surface. To the best of our knowledge, the immunosensor for DXM through competitive PEC method was proposed for the first time. Compared with the reported detection method^26^,^27^,^28^, this assay is more sensitive, inexpensive and time-saving.

## Results and Discussion

### Characterization of the materials

**Figure 2:**
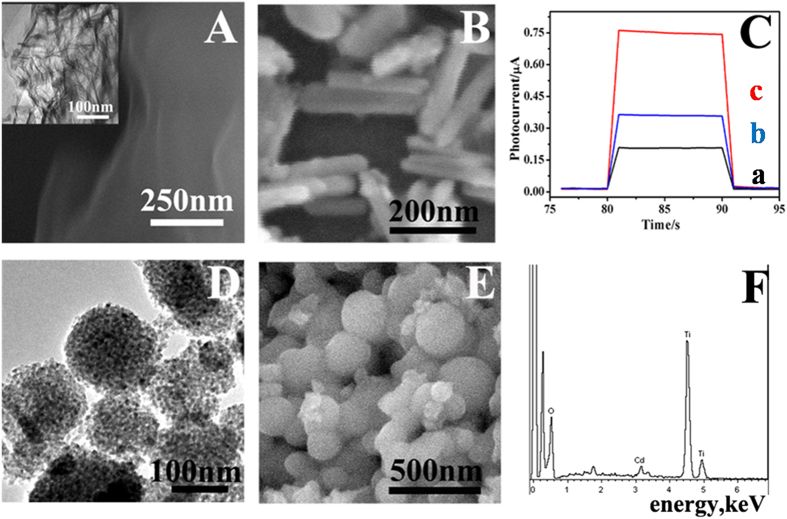
(**A**) The SEM image of carboxylated g-C_3_N_4_. Inset: TEM image of carboxylated g-C_3_N_4_. (**B**) SEM images of Bi_2_S_3_ NRs. (**C**) Transient photocurrents of Bi_2_S_3_ NRs (**a**), g-C_3_N_4_ (**b**), g-C_3_N_4_/Bi_2_S_3_ (**c**). (**D**) TEM image of TiO_2_. (**E**) SEM image and EDS of Cd^2+^@TiO_2_ (**F**).

The morphology of the prepared carboxylated g-C_3_N_4_ was observed by SEM and TEM images respectively in Fig. 2A, illustrating the nanosheets structure composed of large dense thick layers. Figure S1 in the supporting information showed the FT-IR spectrum of g-C_3_N_4_ (line a) and carboxylated g-C_3_N_4_ (line b). The band at 806 cm^−1^ is characteristic peak of tri-s-triazine, and the representative absorption peaks for -COO^–^ at 1386 and 1575 cm^−1^ were found in the line b, indicating the successful functionalization of carboxyl group on the g-C_3_N_4_^29^,^30^. The introduced -COO^-^ of g-C_3_N_4_ is beneficial to uniformly disperse in aqueous solution. As shown in Fig. 2B, the Bi_2_S_3_ NRs structures consisting of some well dispersed nanorods have uniform and small size of about 40 nm in diameter and 200 nm in length. The uniform and small size of the Bi_2_S_3_ NRs is contributed to absorb on the surface of g-C_3_N_4_ and in favor of loading BSA-DXM. Figure S1A showed the structure of g-C_3_N_4_/Bi_2_S_3_, it can be seen that there were rod-like Bi_2_S_3_ dispersed on the surface of g-C_3_N_4_ nanosheets, which were adhered to the substrate stably by annealing. Figure 2C showed that the photocurrent increased obviously after the combination of g-C_3_N_4_ and Bi_2_S_3_ (line c) compared with pure g-C_3_N_4_ (line b) and Bi_2_S_3_ (line a). The enhanced activity is ascribed to the coupled system which offers the effective separation of electron-holes pairs and a wide response wavelength range.

According to the TEM image in Fig. 2D, it is clearly to see the nanoporous structure of TiO_2_, which can provide numerous chemical sorption sites to load a large amount of Cd^2+^ and further for more desired CdS production through the S^2–^ deposition. In addition, as shown in Fig. 2E, the as-synthesized TiO_2_ nanoparticles (NPs) have relatively uniform size with an average diameter of ~200 nm, and the corresponding EDS spectrum showed in the Fig. 2F demonstrated the successful preparation of the Cd^2+^@TiO_2_.

### Characterization of the PEC immunosensor

**Figure 3:**
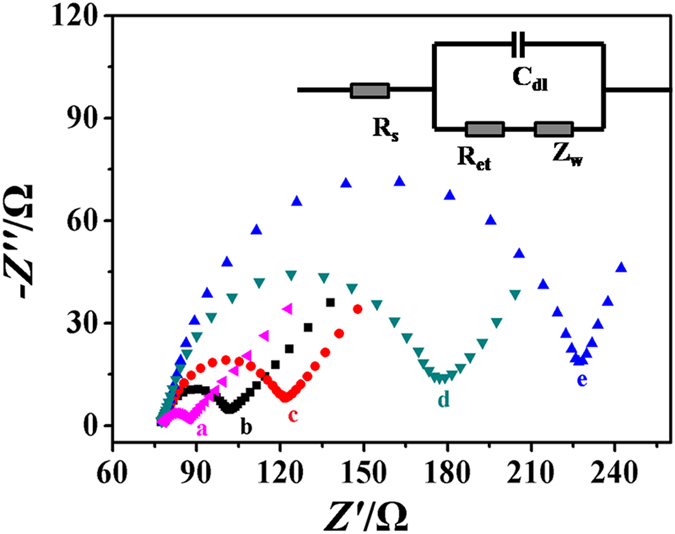
EIS characterization of the sensing interface: (a) bare ITO, (b) ITO/g-C_3_N_4_/Bi_2_S_3_, (c) ITO/g-C_3_N_4_/Bi_2_S_3_/CS, (d) ITO/g-C_3_N_4_/Bi_2_S_3_/CS/DXM/BSA, (e) ITO/g-C_3_N_4_/Bi_2_S_3_/CS/DXM/BSA/Cd^2+^@TiO_2_-anti-DXM. The inset is the Randles equivalent circuit.

Electrochemical impedance spectroscopy (EIS) is an effective approach for characterizing the interface properties of electrodes and demonstrating the full modification process of the immunosensors^31^. Nyquist plots of EIS corresponding to the stepwise modified processes were shown in Fig. 3. The inset is Randles equivalent circuit containing the solution resistance (R_s_), the Warburg impedance (Z_ω_), the double layer capacitance (C_dl_) and the apparent charge transfer resistance (R_et_). The values of them simulated with ZSimWin software and the relevant data were shown in Table S1. Throughout the whole processes of electrode modification, the changes in R_et_ were the most significant among other impedance components. Thus, R_et_ was an appropriate indicator for illustrating the interfacial properties of the modified ITO electrode. For the bare ITO electrode, the impedance spectrum exhibited a small semicircle (curve a), implying a low electron transfer resistance. When the g-C_3_N_4_/Bi_2_S_3_ was assembled and sintered on the surface of ITO electrode, the electron transfer resistance increased apparently (curve b), which was attributed to the g-C_3_N_4_/Bi_2_S_3_ layer inhibited the electron transfer from the electrode to the solution in the system. After the formation of g-C_3_N_4_/Bi_2_S_3_/CS film, the R_et_ value significantly increased (curve c) because of the hindrance effect of CS. Since nonconductive properties of proteins progressively impeded the mass transport and electron transfer to the electrode surface by elevating the hindrance effect and final insulating effect^32^, the R_et_ value gradually increased (curve d, e) after the DXM, BSA and Cd^2+^@TiO_2_-anti-DXM bioconjugates modified on the ITO/g-C_3_N_4_/Bi_2_S_3_/CS electrode surface in turns, which indicated the successful assembling of them. In this assay, the increase of R_et_ value step by step demonstrated the successful fabrication processes of the immunosensor.

SEM images were also used for investigating the surface structure and morphology of the immunosensor. Figure S2B showed the SEM image of ITO/g-C_3_N_4_/Bi_2_S_3_ modified by CS on its framework. As it shown clearly, CS was adhered to the electrode surface. The layered structure of g-C_3_N_4_ greatly widens its surface area so that more CS could adhere to its surface and interior, which is contribute to immobilize more DXM. As shown in Figure S2C, after activation of the CS by glutaraldehyde, the DXM and BSA were immobilized on the film surface through covalent bond formation between amino group and aldehyde group. Figure S2D showed clearly that there were plenty of TiO_2_ NPs dispersed on the electrode surface, which indicating the successful immobilization of Cd^2+^@TiO_2_-anti-DXM bioconjugates on the biosensor surface. According to the above results, the successful fabrication of the PEC biosensor can be proved again.

### The optimization of experimental conditions

**Figure 4:**
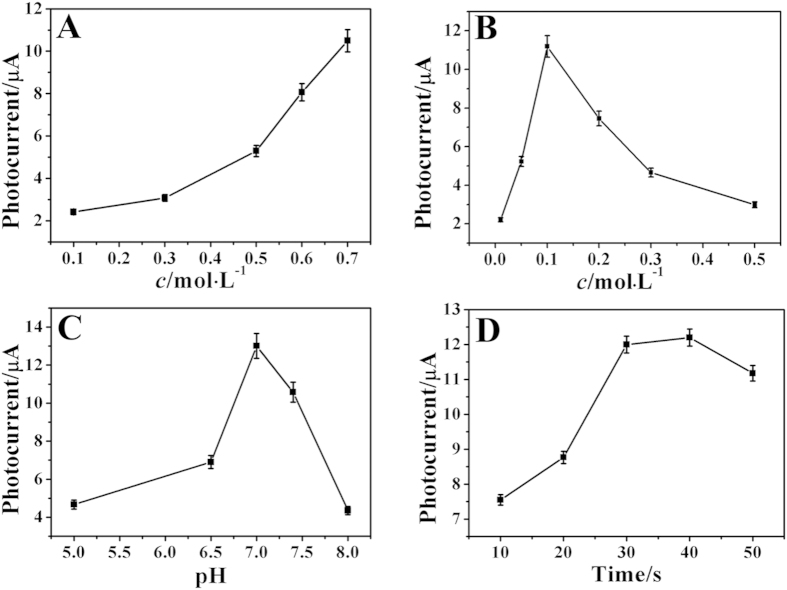
The optimization of experimental parameters: the effect of the concentration of Na_2_S (**A**) and the concentration of AA (**B**) and buffer solution pH (**C**) and the incubation time (**D**) on the photocurrent responses.

In order to obtain the best performance for PEC detection of DXM, the experiment conditions were optimized. In this work, the concentration of Na_2_S is an important influential factor to the photocurrent intensity. As shown in Fig. 4A, the photocurrent responses stepped up gradually with the increase of the Na_2_S concentration from 0.1 to 0.7 mol∙L^−1^, which was attributed to the increasing CdS generated on the surface of electrode. Therefore, 0.7 mol∙L^−1^ of Na_2_S, which is the biggest solubility, was selected as the optimum concentration. In this assay, CdS nanoparticles existed a corrosion process under illumination: 2h^+^ + CdS → Cd^2+^ + S, hence the concentration of AA which was used as an electron donor to suppress the recombination of electrons and holes, also has significant effect on the sensing performance. AA in lower concentrations would lead to a decline in electrical output because fewer reducing agent molecules were available for electron donation to photogenerated holes, while a higher concentration of AA increased the absorbance of the solution which resulted in the decrease of irradiation intensity and efficiency of excited electron-hole center formation^33^. For this reason, as shown in Fig. 4B, the photocurrent increased with increasing AA concentration up to 0.1 mol∙L^−1^, and then decreased for much higher concentrations of AA. Thus, 0.1 mol∙L^−1^ was chosen as the optimum concentration of AA for the following test. Figure 4C showed the influence of pH (5.0 ~ 8.0) on the photocurrent responses of the immunosensor. Obviously, the immunosensor showed the best performance at pH 7.0. The reason might be the biological activity of protein was damaged when the environment is overly acidic or basic. As a result, pH 7.0 was employed as the optimal condition. In addition, the effect of incubation time on the response of the sensing system was also evaluated. As shown in Fig. 4D, the photocurrent responses took place small change when the time more than 30 min. Thus, 30 min was selected as the optimal incubation time in this experiment.

### Analytical performance

**Figure 5:**
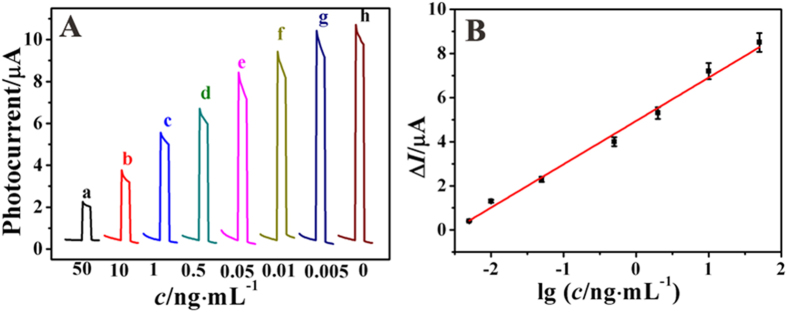
(**A**) Time-based photocurrent responses of the immunosensor incubated with various concentrations of DXM, from (a–h) are 50, 10, 1, 0.5, 0.05, 0.01, 0.005, 0 ng∙mL^−1^, respectively. (**B**) The corresponding calibration curve between the photocurrent change (ΔI) and various DXM concentrations.

The competitive PEC immunosensor was applied to detect various concentrations of dexamethasone under the optimized conditions. The target DXM competed with the immobilized DXM on the electrode surface to bind the limited binding sites of the Cd^2+^@TiO_2_-anti-DXM to form the immunocomplexs. With the increase of target DXM, the amount of CdS *in situ* generated on the electrode accordingly decreased. Thus, as shown in Fig. 5, the photocurrent response intensity was proportional to the concentration logarithm of target DXM in the range of 5 pg∙mL^−1^ to 50 ng∙mL^−1^ with a correlation coefficient of 0.982 and the limit of detection (LOD) was estimated to be 2 pg∙mL^−1^ (S/N = 3). Compared with the previous reports, the analytical performance of our PEC sensor showed superiority over other methods in terms of both detection limit and dynamic range, as shown in Table S2 in the Supporting information.

### Reproducibility, selectivity and stability of the PEC immunosensor

To examine the reproducibility of the biosensor, the photocurrent responses of five different modified electrodes prepared in the parallel experimental conditions to 1.0 ng∙mL^−1^ of DXM were investigated. Relative standard deviation (RSD) of 3.17% was obtained, which indicated the preferable precision and acceptable reproducibility of the immunosensor.

**Figure 6:**
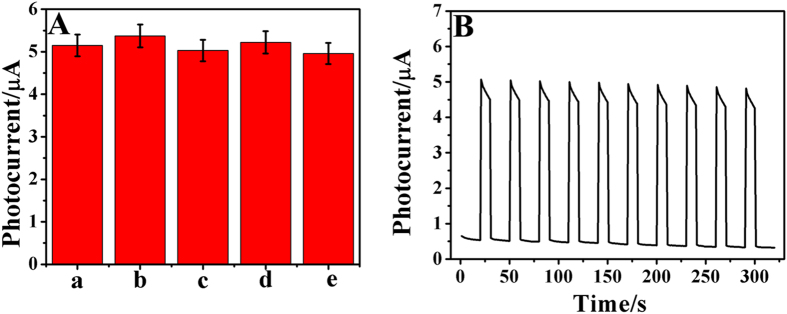
(**A**) The responses of interference. (a) 1.0 ng∙mL^−1^ DXM, (b) 1.0 ng∙mL^−1^ DXM + 100 ng∙mL^−1^ BSA, (c) 1.0 ng∙mL^−1^ DXM + 100 ng∙mL^−1^ glucose, (d) 1.0 ng∙mL^−1^ DXM + 100 ng∙mL^−1^ aflatoxin M1, (e) 1.0 ng∙mL^−1^ DXM + 100 ng∙mL^−1^ ochratoxin A. (**B**) Time based photocurrent response of the immunosensor repeated every 10 s under several on/off irradiation cycles for 300 s.

The selectivity of the immunosensor was evaluated by testing the photocurrent responses of DXM (1.0 ng∙mL^−1^) containing 100 ng∙mL^−1^ BSA, glucose, aflatoxin M1 and ochratoxin A. The results are shown in Fig. 6A. It was obvious that the developed biosensor has good specificity to DXM.

The stability was also an important standard to test the performance of the immunosensor. As shown in Fig. 6B, no apparent variation of reproducible photocurrent response was recorded under 10 times on/off irradiation cycles, which implied the excellent stability of the proposed assay.

### Application in analysis of samples

The practical application of the immunoassay was investigated by analyzing the recoveries of different concentrations of DXM in milk samples. Different concentrations (1.00, 5.00 and 10.0 ng∙mL^−1^) of DXM spiked milk samples were prepared by standard addition methods. The recoveries of the immunosensor were in the range of 99.4 ~ 108% and the relative standard deviation (RSD) was 2.22 ~ 3.56% (Table 1), indicating that the proposed immunosensor could be applied to the determination of DXM in real samples.

## Conclusion

In summary, a competitive PEC biosensor for the rapid and sensitive determination of DXM under visible light irradiation has been developed based on a novel signal amplification strategy. The PEC immunosensor was designed using carboxylated g-C_3_N_4_ coupled with Bi_2_S_3_ which were first used for fabricating PEC immunosensor as substrate, and the Cd^2+^@TiO_2_ was used as the label to generate CdS *in situ* for the signal amplification. The heterostructure of g-C_3_N_4_/Bi_2_S_3_ improved the photocurrent intensity effectively due to the enhanced charge separation. Moreover, the favorable matching of energy levels between TiO_2_ and CdS also in favor of improving the detection sensitivity. This well-designed immunosensor was easy to fabricate and exhibited rapid, sensitive and highly selective detection for DXM.

## Materials and Methods

### Chemicals and reagents

ITO (resistivity 10 Ω/sq) was purchased from Zhuhai Kaivo Electronic Components Co. Ltd., China. Tetrabutyl titanate (TBOT), glutaraldehyde, poly (allylamine hydrochloride) (PAH), ethylene glycol, bismuth nitrate hydrate (Bi(NO_3_)_3_∙5H_2_O), cadmium nitrate tetrahydrate (Cd(NO_3_)_2_∙4H_2_O) and sodium sulfide (Na_2_S) were purchased from Shanghai Chemical Reagent Co. Ltd., (Shanghai, China). DXM and anti-DXM were obtained from Beijing Biosynthesis Biotechnology Co. Ltd., (Beijing, China). Chitosan (CS), bovine serum albumin (BSA) and ascorbic acid (AA) were obtained from Sigma-Aldrich. Phosphate buffer saline (PBS) was prepared by using 0.1 mol∙L^−1^ Na_2_HPO_4_ and 0.1 mol∙L^−1^ KH_2_PO_4_ solution. All aqueous solutions were prepared using ultrapure water.

### Apparatus

Transmission electron microscope (TEM) images were obtained from an H-800 microscope (Hitachi, Japan). Scanning electron microscope (SEM) images and Energy Dispersive X-Ray Spectroscopy (EDS) were recorded by JEOL JSM 6700F microscope (Japan). Fourier Transform Infrared Spectrometer (FT-IR) was examined with a VERTEX70 spectrometer (Bruker Co. Ger.). Photocurrent was measured by the current-time curve experimental technique on an electrochemical workstation (Zahner Zennium PP211, Germany) at a bias voltage of 0.1 V with light intensity of 200 W cm^−2^. All experiments were carried out at room temperature using a conventional three electrode system. Electrochemical impedance spectroscopy (EIS) was performed on an autolab potentiostat/galvanostat (Zahner, Germany) with a three electrode system.

### Synthesis of Bi_2_S_3_ NRs

The synthesis of Bi_2_S_3_ NRs is based on the previous report^34^. First of all, 1.82 g Bi(NO_3_)_3_∙5H_2_O was dissolved into 25 mL ethylene glycol and deaerated by nitrogen bubbling for 15 min (Defined as solution A). At the same time, 1.35 g Na_2_S was dissolved into the mixture containing 10 mL ethylene glycol and 20 mL ultrapure water. After stirred for 15 min, solution B was obtained. Then, solution B was added into solution A dropwise under magnetic stirring, following with amount of blank suspension appeared. 1.92 g carbamide and 20 mL ultrapure water were added into the mixture continuing stirring for another 30 min. Subsequently, the solution was transferred into autoclave which was heated 180 °C for 24 h. When the reaction was cooled to room temperature, the precipitate was collected by vacuum filtration, then centrifuged and washed with ethanol and ultrapure water for several times to remove unnecessary chemicals. Finally, the Bi_2_S_3_ NRs were obtained after dried under vacuum at 60 °C.

### Synthesis of Cd^2+^@TiO_2_ and Cd^2+^@TiO_2_-anti-DXM conjugates

The TiO_2_ was synthesized according to the previous report^35^. Briefly, 0.01 mol∙L^−1^ TBOT was added into 15 mL ethanol and stirred for 2 h. Then the solution was transferred into a 50 mL autoclave and heated at 200 °C for 10 h. Afterward, the obtained white precipitates were collected and washed thoroughly by ultrapure water and ethanol for several times to remove the unnecessary chemicals, and then dried in vacuum at 80 °C for 12 h. To obtain Cd^2+^@TiO_2_, 20 mg TiO_2_ NPs were dispersed in 17 mL Cd(NO_3_)_2_∙4H_2_O (10 mmol∙L^−1^) aqueous solution and stirred at 50 °C for 2 h. The resulted hybrid nanoparticles were extracted by centrifugation and washed with distilled water.

For synthesis Cd^2+^@TiO_2_-anti-DXM bioconjugates, firstly, Cd^2+^@TiO_2_ hybrid nanoparticles were dispersed in 7.5 mL of PAH (2 mg∙mL^−1^) aqueous solution and stirred for 1 h. After that, the solution was centrifuged and washed with ultrapure water, and then the obtained nanoparticles were redispersed in 200 μL of glutaraldehyde (wt. 2.5%) and oscillated for 4 h. After centrifuging and washing with distilled water, the hybrid nanoparticles were added into 100 μL of anti-DXM (100 μg∙mL^−1^) solution and incubated for 20 h under shaking at 4 °C, followed by centrifugation to remove the free anti-DXM. Finally, the Cd^2+^@TiO_2_-anti-DXM conjugates were dispersed in 2 mL of PBS (pH 7.0) which containing 1% BSA and stored in 4 °C for future use.

### Fabrication of the PEC immunosensor

Before preparation, ITO glasses were sonicated in acetone, ethanol and ultrapure water in turns for about 30 min each, and then blew dry under the stream of nitrogen. First, 6 μL of carboxylated g-C_3_N_4_ and Bi_2_S_3_ solution were coated onto ITO electrode in turns and dried at room temperature, then the electrode was calcined to ensure particles adhesion to the substrate stably at 400 °C for 30 min in air. After cooled down to room temperature, 6 μL CS solution (1%) was spread on the surface of ITO/g-C_3_N_4_/Bi_2_S_3_ electrode, obtaining the ITO/g-C_3_N_4_/Bi_2_S_3_/CS electrode. Subsequently, 6 μL of BSA-DXM was dropped on the electrode surface and incubated for 60 min at 4 °C, followed by rinsing with the washing buffer. After reaction for 50 min, the nonspecific binding sites were blocked by adding 3 μL of BSA (wt. 1%) and then washed with the washing buffer thoroughly. For the competitive immune recognition reaction, first, a certain concentration of Cd^2+^@TiO_2_-anti-DXM conjugate was mixed with the incubation solution containing a standard solution of DXM with a series of known concentrations. Then, 6 μL of the mixing solution was added onto electrode surface and incubated for another 30 min at room temperature to facilitate the competitive immunoassay. Finally, for the purpose of *in situ* generated CdS-enhanced photocurrent, 6 μL of Na_2_S solution (0.7 mol∙L^−1^) was added and incubated for 5 min at room temperature in the dark and then washed by washing buffer solution. So far, the competitive PEC immunosensor was constructed successfully.

## Additional Information

**How to cite this article**: Wang, X. *et al.* A competitive photoelectrochemical immunosensor based on a CdS-induced signal amplification strategy for the ultrasensitive detection of dexamethasone. *Sci. Rep.* **5**, 17945; doi: 10.1038/srep17945 (2015).

## Supplementary Material

Supplementary Information

## Figures and Tables

**Figure 1:**
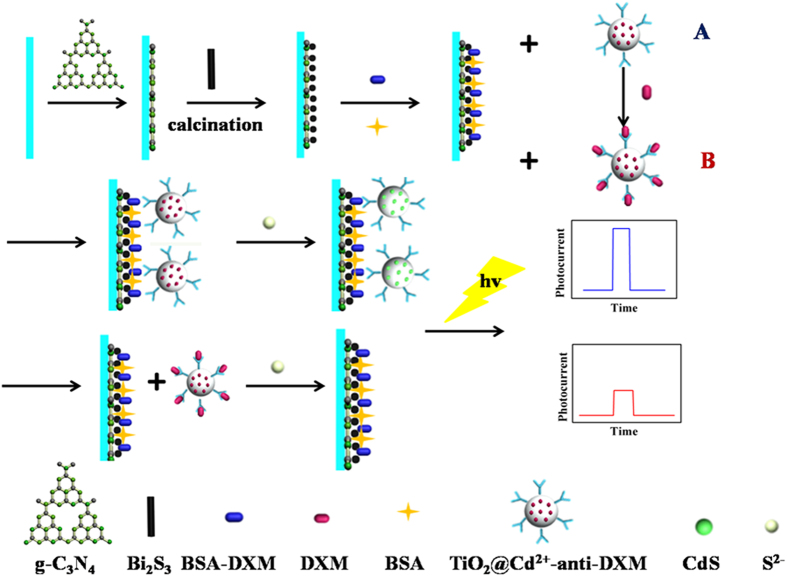
Schematic illustration of the fabrication process of the competitive sensing platform. Process (**A**) and (**B**) respectively represents the preparation of electrode modified with Cd^2+^@TiO_2_-anti-DXM and Cd^2+^@TiO_2_-anti-DXM containing DXM of different concentration. Under light illumination, the changed current due to various amounts of Cd^2+^@TiO_2_-anti-DXM immobilized on electrode surface is recorded as the sensing signal.

**Table 1:** Detection results of DXM-spiked milk samples.

Addition content (ng∙mL^−1^)	The detected content (ng∙mL^−1^)	Average value (ng∙mL^−1^)	RSD (%, n = 5)	Recovery (%, n = 5)
1.00	1.11, 1.08, 1.03,1.12, 1.05	1.08	3.56	108
5.00	4.87, 4.93, 5.15, 4.81, 5.08	4.97	2.87	99.4
10.0	10.35, 9.87, 10.06, 10.42, 10.27	10.2	2.22	102
