# Supplementary material for: A competitive photoelectrochemical immunosensor based on a CdS-induced signal amplification strategy for the ultrasensitive detection of dexamethasone
Source: Sci Rep. 2015 Dec 9;5:17945. doi: 10.1038/srep17945 (PMC4673695; doi:10.1038/srep17945)
Supplement: Supplementary Information [file srep17945-s1.doc]

*Supplementary Materials*

**A competitive photoelectrochemical immunosensor based on a CdS-induced signal amplification strategy for the ultrasensitive detection of dexamethasone**

Xueping Wang^a^, Tao Yan^b^, Yan Li^a^, Yixin Liu^a^, Bin Du^b^, Hongmin Ma^a^,*, Qin Wei^a^,*

^a^Key Laboratory of Chemical Sensing & Analysis in Universities of Shandong, School of Chemistry and Chemical Engineering, University of Jinan, Jinan 250022, PR China

^b^School of Resources and Environment, University of Jinan, Jinan 250022, PR China

^*^Corresponding author. Tel: + 86 531 82767872; fax: + 86 531 82767367.

E-mail address: mahongmin2002@126.com (Hongmin Ma);

sdjndxwq@163.com (Qin Wei).

**Synthesis of the** **carboxylated g-C_3_N_4_ nanosheets**

The carboxylated g-C_3_N_4_ was synthesized according to the previous report with slight modification.[^1^](#_ENREF_1) In brief, white melamine powder was placed into a covered ceramic crucible and heated at 550 ℃ for 4 h in a muffle furnace. After cooling to room temperature naturally, the yellow g-C_3_N_4_ product was ground to powder for further use. Then, 1 g g-C_3_N_4_ powder was put into 100 mL 5 mol∙L^-1^ HNO_3_ and refluxed for 24 h at 125 ℃. After cooling to the room temperature naturally, the product was centrifuged and washed with ultrapure water until pH value reached 7.0 and vacuum dried at 35 ℃ for 12 h. The desired carboxylated g-C_3_N_4_ was obtained.


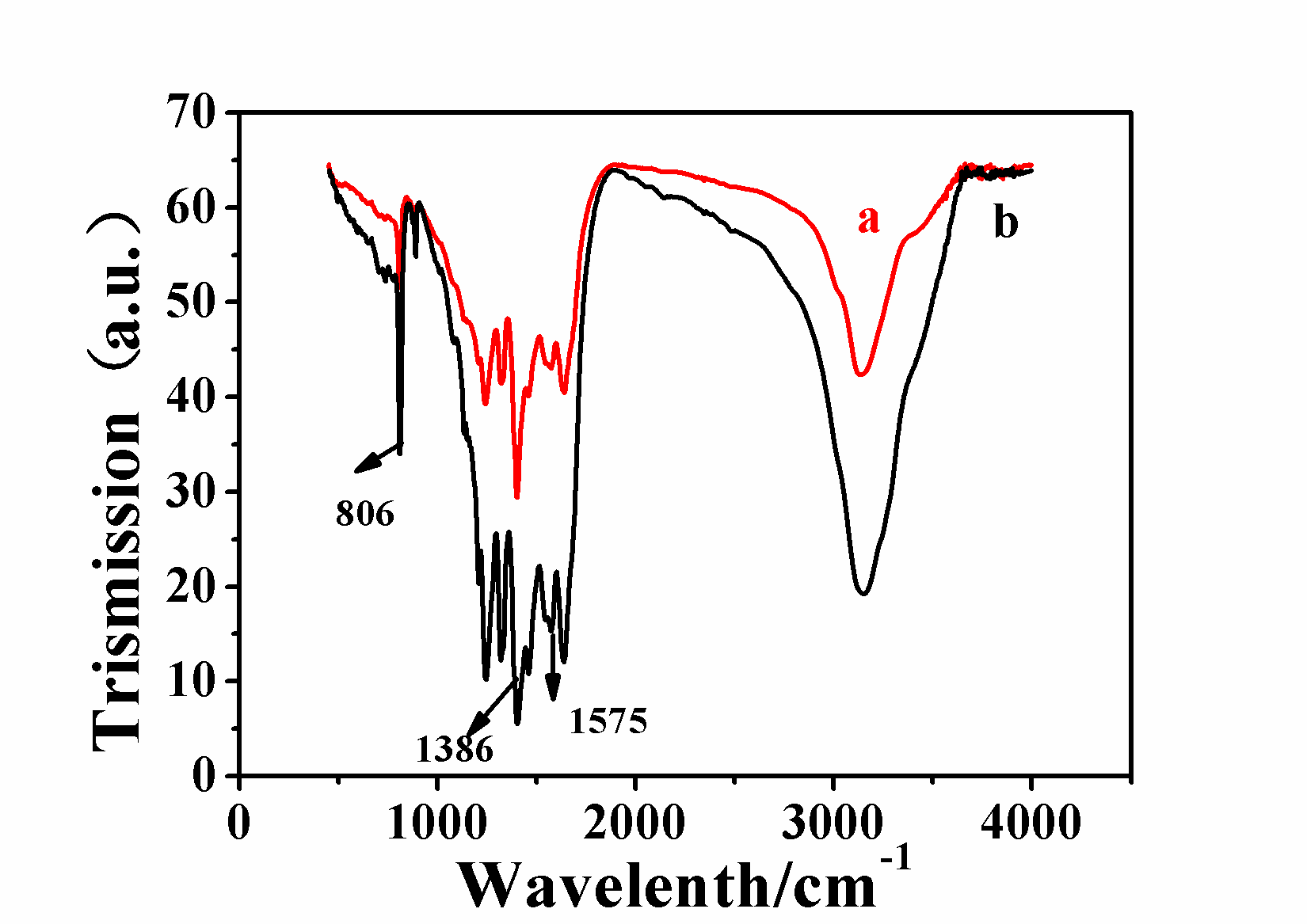


Figure S1. FT-IR spectroscopy of g-C_3_N_4_ (a) and carboxylated g-C_3_N_4_ (b).


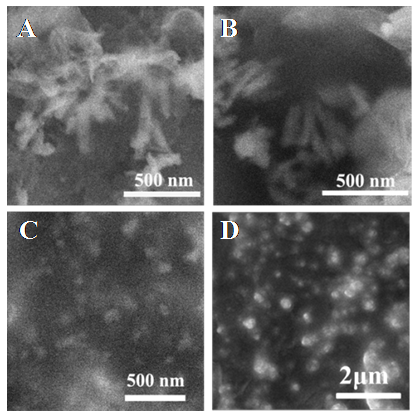


Figure S2. The SEM images of modified ITO electrode: (a) ITO/g-C_3_N_4_/Bi_2_S_3_, (b) ITO/g-C_3_N_4_/Bi_2_S_3_/CS, (c) ITO/g-C_3_N_4_/Bi_2_S_3_/CS/DXM/BSA, (d) ITO/g-C_3_N_4_/Bi_2_S_3_/CS/DXM/BSA/Cd^2+^@TiO_2_-anti-DXM.

Table S1 Simulation parameters of the equivalent circuit components

| Electrode | *R*_s_ (Ω) | *R*_et_ (Ω) | *C*_dl_ (F) | Z_W_ |
| --- | --- | --- | --- | --- |
| ITO | 79.26 | 7.757 | 1.355×10^-5^ | 0.01397 |
| ITO/g-C_3_N_4_/Bi_2_S_3_ | 78.87 | 22.04 | 1.047×10^-5^ | 0.01316 |
| ITO/g-C_3_N_4_/Bi_2_S_3_/CS | 81.09 | 38.37 | 9.297×10^-6^ | 0.01183 |
| ITO/g-C_3_N_4_/Bi_2_S_3_/CS/DXM/BSA | 81.04 | 90.02 | 7.334×10^-6^ | 0.01032 |
| ITO/g-C_3_N_4_/Bi_2_S_3_/CS/DXM/BSA/Cd^2+^@TiO_2_-anti-DXM | 79.55 | 138.1 | 4.832×10^-6^ | 0.01226 |

Table S2 Analytical performance of different methods for dexamethasone detection

| Analytical methods | Linear range | Limit of detection | Reference |
| --- | --- | --- | --- |
| Electrochemical method | 1 nM~100 μM | 9.1×10^-10^ M | [^2^](#_ENREF_2) |
| chemiluminescence method | 0.004~25.0 mg∙L^-1^ | 0.0013 mg∙L^-1^ | [^3^](#_ENREF_3) |
| liquid chromatography-atmospheric pressure chemical ionization-tandem mass spectrometric method | 0.15~5 ng∙L^-1^ | 41 pg∙mL^-1^ | [^4^](#_ENREF_4) |
| ultraviolet spectrophotometric method | 1~30 µg∙mL^-1^ | 0.52 µg∙mL^-1^ | [^5^](#_ENREF_5) |
| the proposed method | 0.005 ~50 ng∙mL^-1^ | 0.002 ng∙mL^-1^ | This work |

1. Cheng, C. et al. Anodic electrogenerated chemiluminescence behavior of graphite-like carbon nitride and its sensing for rutin. *Anal. Chem.* **85**, 2601-2605 (2013).

2. Goyal, R.N., Chatterjee, S. & Rana, A.R.S. Effect of cetyltrimethyl ammonium bromide on electrochemical determination of dexamethasone. *Electroanal.* **22**, 2330-2338 (2010).

3. Khataee, A., Hasanzadeh, A., Lotfi, R., Pourata, R. & Joo, S.W. Determination of dexamethasone by flow-injection chemiluminescence method using capped CdS quantum dots. *Spectrochim. Acta A.* **150**. 63-71 (2015).

4. Cherlet, M., De Baere, S. & De Backer, P. Quantitative determination of dexamethasone in bovine milk by liquid chromatography-atmospheric pressure chemical ionization-tandem mass spectrometry. *J. Chromatogr. B* **805**, 57-65 (2004).

5. Friedrich, R.B., Ravanello, A., Cichota, L.C., Rolim, C.M.B. & Beck, R.C.R. Validation of a simple and rapid UV spectrophotometric method for dexamethasone assay in tablets. *Quím. Nova* **32**, 1052-1054 (2009).
